# What predicts preservice teachers' classroom management self-efficacy? A cross-cultural comparison between China and Australia

**DOI:** 10.3389/fpsyg.2026.1892257

**Published:** 2026-08-12

**Authors:** Siyu Duan, Kerry Bissaker

**Affiliations:** 1 Department of Teacher Development, Chongqing Academy of Education Science, Chongqing, China; 2 College of Education, Psychology and Social Work, Flinders University, Adelaide, SA, Australia

**Keywords:** classroom management self-efficacy, measurement invariance, preservice teachers, subsampling approach, teacher self-efficacy (TSE)

## Abstract

This research examines predictors of preservice teachers' classroom management self-efficacy (CMSE). Our sequential mixed methods research involved cohorts of final year preservice teachers enrolled in either Chinese or Australian universities. Findings show that CMSE is shaped more by experience-based factors—such as perceived support during school-based placements, satisfaction with course content, and the perceived relevance of prior experience—than by university-based coursework participation. Perceived support mitigated negative experiences, although Chinese preservice teachers reported lower levels of perceived support than Australian peers. Satisfaction with course content highlighted the importance of perceived quality and applicability, while prior experience contributed only when interpreted as relevant. Qualitative findings further indicate that adaptive coping strategies shape how negative experiences are processed, underscoring the role of interpretation in CMSE development. These findings carry important implications for initial teacher education.

## 1 Introduction

Self-efficacy is a self-perception of one's capacity to accomplish a certain task and decades of research has demonstrated that self-efficacy in a specific field significantly influences an individual's performance within that field (Bandura, 1977). Classroom management self-efficacy (CMSE) refers to perception of one's personal ability to effectively manage classroom dynamics, including controlling disruptive behaviors, calming and addressing defiant students, and establishing routines and order, to ensure that learning activities proceed smoothly (Aloe et al., 2014). Teachers, especially novice teachers, consistently report low confidence and feelings of being unprepared for managing their classrooms within China (Liu, 2019; Xu, 2015), Australia (Mayer et al., 2017), and internationally (Dicke et al., 2014; Kee, 2011; Kwok and Svajda-Hardy, 2021). The importance of classroom management (CM), particularly for preservice teachers (PSTs) and graduate teachers, continues to attract the attention of scholars focusing on teacher self-efficacy (TSE) in the area of CM (Aloe et al., 2014; Duan et al., 2024; Hettinger et al., 2021).

Self-efficacy beliefs are most malleable early in learning and are rather stable once established (Bandura, 1997). Numerous TSE researchers focus on PST and early career teachers in seeking to understand influences on the formation of TSE beliefs (Chiang, 2008; Gurvitch and Metzler, 2009). Bandura (1997) posited that individuals assess and judge their self-efficacy by interpreting information from four sources: mastery experiences, vicarious experiences, social and verbal persuasions, and physiological and affective states. While prior quantitative research has often operationalised self-efficacy sources using composite scales (e.g., Clark and Newberry, 2019; Pfitzner-Eden, 2016), the conceptualization and measurement of these sources remain inconsistent across studies (Morris et al., 2017). As a result, it remains unclear how specific experiences and contextual factors contribute to PSTs' CMSE. Accordingly, this research focused on how proximal features of PSTs' actual experiences—such as perceived support during school-based placements, university-based coursework participation—shape CMSE.

The construct of TSE is largely rooted in western countries so various research emphasizes the need for cross-cultural studies of TSE (Klassen et al., 2010; Vieluf et al., 2013). Although the universal benefits of TSE (i.e., how it affects student educational outcomes) has been demonstrated both theoretically (Bandura, 1995) and empirically (Scherer et al., 2016; Yada et al., 2018), the sources of self-efficacy information varies in different cultural contexts (Bandura, 1995). However, the developments of TSE among different contexts are rarely compared, so in response, this study examined the predictors of PSTs' CMSE in one east Asian country (China) and one Anglo-Saxon country (Australia).

### 1.1 Measuring CMSE

Since the early 1980s, TSE scales have included items related to CM. For example, one CM item from Gibson and Dembo (1984) instrument stated:

“*If a student in my class becomes disruptive or noisy, I feel assured that I know some techniques to redirect him quickly.”*

Emmer and Hickman (1991) were among the first to develop a sub-scale specifically designed to capture teachers' belief in the domain of CM. They modified the widely adopted Gibson and Dembo (1984) scale by including additional items related to CM and discipline. The well-known Teachers' Sense of Self-Efficacy Scale (TSES) by Tschannen-Moran and Hoy (2001) assesses three dimensions of teaching self-efficacy: self-efficacy for student engagement, self-efficacy for instructional strategies, and self-efficacy for classroom management. An example item for the CM dimension is:

“*How much can you do to get children to follow classroom rules?”*

With the establishment of CM as a distinct sub-component of TSE, scales were also designed to solely assess CMSE as a single domain. For example, to assess teaching self-efficacy (TSE), Betoret (2009) employed two scales: Teacher-perceived teaching self-efficacy (10 items) and Teacher-perceived self-efficacy in classroom management (4 items). Using a sample of PSTs, Main and Hammond (2008) also developed a 14-item Behavior Management Self-Efficacy Scale, which was adapted from Brouwers and Tomic (1999). The acknowledgment of CM as a distinct teaching domain has seen the rapid expansion of the measurement of teachers' and PSTs' CMSE with CMSE frequently assessed using the sub-scale of Tschannen-Moran and Hoy (2001) instrument (Duan et al., 2024).

### 1.2 Preservice teachers' CMSE development

Perhaps due to the well-established benefits of the TSE/CMSE construct (Dicke et al., 2014; Ryan et al., 2015), TSE/CMSE has been viewed as an outcome of initial teacher education (ITE) courses (Rots et al., 2014, 2007). The impact of ITE programs (university-based coursework and school-based placement) on PSTs' self-efficacy are frequently examined. For example, Main and Hammond (2008) found actual classroom experience enhanced PSTs' self-efficacy beliefs for behavior management by investigating SE beliefs before and after their practicum experiences. O'Neill and Stephenson (2012b) investigated the number of topics that focused on CM but found that completion of coursework did not contribute to PSTs' CMSE. In addition to formal experience, Tuchman and Isaacs (2011) also suggested that the formative experiences of TSE should include informal practice experience outside of classrooms. In their study, experiences as a youth advisor, as a camp counselor and as a childcare supervisor were all found to be positively associated with TSE. Theoretically, informal experience outside the classroom is a vital form of *enactive mastery experience* (Bandura, 1997).

Beyond the ITE program elements, personality characteristics have also been identified as important influences on TSE/CMSE (Baranczuk, 2021). As noted by Yeh (2006), “a greater emphasis should be placed on the nurturing of certain personal traits, especially those that involve the monitoring and development of the cognitive processes required in teaching situations” (p. 514), to help teachers build TSE. Baranczuk (2021) conducted a meta-analysis to synthesize the association between the Big Five personality traits and TSE and included 53 studies in the meta-analysis. The result showed that lower neuroticism and higher extraversion, openness, agreeableness, and conscientiousness were related to higher level of TSE. However, a more recent systematic review by Duan et al. (2024) found openness, extraversion and conscientiousness related to CMSE, whereas agreeableness and neuroticism had no relationship with CMSE. The inconsistent results between global TSE and domain-specific TSE highlights the importance of examining the special characteristics of CMSE.

### 1.3 Cross-cultural perspectives

Cross-cultural studies on TSE primarily focus on validating its factorial structure (e.g., Fackler et al., 2020; Vieluf et al., 2013) and demonstrating its positive impact on the educational process and outcomes (e.g., Klassen et al., 2009; Scherer et al., 2016). As Bandura (2002) argued, “there is commonality in basic agentic capacities and mechanisms of operation, but diversity in the culturing of these inherent capacities” (p. 273). TSE/CMSE has been well established as an essential psychological construct influencing teaching and learning in different cultural groups. However, cultural factors may influence the informative process of CMSE and limited research has focused on the similarities and differences in the development of CMSE across different contexts, especially for PSTs.

While cross-cultural TSE/CMSE research helps to strengthen the validity and generalizability of TSE theory (Klassen et al., 2011), there are some challenges in achieving a valid comparison between different groups. Comparison cannot be made if an instrument measures different constructs in two cultures (Vijver, 2021). Although self-efficacy is widely recognized across the globe, educational concepts such as “classroom management” or “behavior management” may have different meanings between China and Australia (Lewis et al., 2005). In cross-cultural studies, bias could also arise during data collection, i.e., methods bias (Vijver, 2021). For example, response style in Likert scales such as extremity and midpoint responding may cause biases in cross-cultural comparison (Vieluf et al., 2013). The East-Asian cultural norm of modesty may contribute to the relatively stronger tendency toward midpoint responding in these cultures (Chen et al., 1995).

Although it is impossible to eliminate all bias in cross-cultural research, establishing equivalence before comparison is fundamental for conducting rigorous and valid cross-cultural comparisons (Vijver, 2021). Establishing equivalence ensures that the differences observed between groups are due to actual differences in the constructs being measured, rather than differences in how the groups interpret or respond to the measurement. Measurement invariance (Chen et al., 2005) is commonly tested to determine whether the same construct has been measured across cultures in the same way. Such practice is undertaken in many cross-cultural TSE comparisons (e.g., Fackler et al., 2020; Vieluf et al., 2013; Yada et al., 2019, 2018).

### 1.4 Research questions

While numerous researchers have studied TSE, only a few have solely focused on specific facets of TSE (Aloe et al., 2014; Hettinger et al., 2021; Lazarides et al., 2020), and still fewer have compared CMSE across different cultural contexts. This study addresses these research gaps by answering the following questions:

- To what extent do the measures of classroom management self-efficacy and its predictors demonstrate cross-cultural equivalence across the Chinese and Australian cohorts?
- What factors predict preservice teachers' classroom management self-efficacy, and how do these relationships differ between the Chinese and Australiancohorts?

## 2 Methods

This cross-cultural study adopted “QUAN → qual” design, a sequential explanatory mixed methods design (Creswell and Clark, 2018). Quantitative survey data were first collected to examine PSTs' CMSE and its predictors across the Chinese and Australian cohorts, with statistical analyses used to identify cross-cultural similarities and differences. Subsequently, qualitative interviews were conducted to provide explanatory insights into why certain predictors, if any, contributed differently to CMSE development. Integrating quantitative and qualitative data can enable a more contextualized understanding of cross-cultural patterns (Schrauf, 2017).

### 2.1 Participants and procedure

Participants were the final year PSTs in China and Australia who were soon to be graduate teachers at K-12 mainstream schools. Specifically, one university in Australia and three universities in China were approached to participate, selecting PSTs who were available and volunteered to participate in the survey and the follow-up interview.

In total, the survey data collection lasted around 3 months (July 2023-September 2023), resulting in 624 samples, where 527 were Chinese PSTs and 97 were Australian PSTs. Details of survey participants from both countries are presented in Table 1. Among 624 PSTs, 94 volunteered for interviews; 18 (9 Chinese, 9 Australian) were purposefully selected to participate in the follow-up interview. To maximize variation, we sampled six participants at each CMSE level (low, medium, high) using the CM subscale of the TSES (Tschannen-Moran and Hoy, 2001).

**Table 1:** Survey participants' demographic information.

Demographic characteristics		China	Australia
		*N* = 527	*N* = 97
Gender	Male	21.60%	15.50%
	Female	78.40%	84.50%
	Non-binary	0	0
	Prefer not to say	0	0
Age	18-22 (school leaver)	79.70%	24.70%
	23-32 (early mature entry)	20.30%	59.80%
	Above 32 (later mature entry)	0.00%	15.50%
School level of placement	Kindergarten	2.70%	29.90%
	Primary school	26.40%	29.90%
	Secondary school	71.00%	40.20%
Classroom management coursework	Yes	89.60%	51.50%
	No	10.40%	36.10%
	Missing	0	12.40%
Experience of interactions with school-age children outside the classroom	Yes	53.50%	69.10%
	No	46.50%	30.90%

### 2.2 Data collection

#### 2.2.1 Multi-scale survey

A multi-scale survey was designed including items covering PSTs' age, gender and level of teaching focus (early childhood, primary or secondary). In terms of PSTs' CMSE formative experience, it was accessed via three easy-to-answer questions related to whether they had completed or were completing course topics focusing primarily on classroom and behavior management theory, knowledge, skills, or strategies (formal experience); their satisfaction with CM content provided in ITE program (formal experience); and whether they had experiences of behavior management with school-aged students outside the classroom, for example sports coaching, tutoring (informal experience). The final item related to their interest in participating in a follow-up interview.

To measure PSTs' CMSE, the 24-item Teachers' Sense of Self-Efficacy Scale (TSES) (Tschannen-Moran and Hoy, 2001) was used. The TSES has both long form (24 items) and short form (12 items). The TSES comprises three sub-scales: instructional strategies (eight/four items), student engagement (eight/four items), and classroom management (eight/four items). All items were rated on a nine-point Likert-type scale, ranging from “Nothing (1)” to “A great deal (9)”. TSES has been tested with good evidence of reliability and validity cross-culturally (Klassen et al., 2009). The Chinese version of TSES has also demonstrated high internal consistency coefficients for both the overall scale and its sub-scales (Minghui, 2017).

PSTs' perceptions of support during the placement were accessed by a perceived support scale (Weber et al., 2019). Participants judged these three statement: *In the event of difficulties, I was able to call on meaningful help at any time; I was shown concrete possibilities for improvement; and I received clear and detailed feedback on my performance*, on a 4-point scale ranging from “does not apply” (1) to “applies” (4). To our knowledge, the perceived support scale has not been tested in China. The perceived support scale was translated into Chinese. To ensure accuracy, back-translation (Brislin, 1970) was used to clarify each item wording.

The 10-item Big Five Inventory (BFI-10) (Rammstedt and John, 2007) was used to measure PSTs' personality traits. BFI-10 is a 5-point scale, ranging from “strongly disagree (1)” to “strongly agree (5)”. Rammstedt and John (2007) reported good evidence of reliability and validity for this scale, including five personality traits: extraversion (2 items); agreeableness (2 items); conscientiousness (2 items); neuroticism (2 items); openness (2 item). The BFI-10 has also been translated and tested in China (Carciofo et al., 2016; Park et al., 2022).

Preliminary analyses indicated that the main constructs demonstrated acceptable reliability across country groups, with Cronbach's alpha coefficients generally exceeding 0.7. However, the BFI-10 subscales showed poor reliability, particularly in the Australian sample and were therefore excluded from subsequent analyses (see details in appendix A). However, the follow-up interview provided valuable insights into the relationship between personality traits and CMSE, which will be explored in the discussion section.

#### 2.2.2 Interview guide

The follow-up interview questions were informed by the survey findings and existing literature on TSE/CMSE. Specifically, the interview focused on three areas: (a) PSTs' perceptions of their CMSE, (b) their school-based placement experiences related to CM, and (c) their university-based coursework learning experiences in CM.

### 2.3 Data analysis

#### 2.3.1 Quantitative analyses

Statistical analysis was performed using IBM SPSS statistics, and Mplus software (Muthén and Muthén, 1998–2012). Given the presence of missing data and non-normality, analyses were conducted using maximum likelihood estimation with robust standard errors (MLR). No missing data were observed in the Chinese sample, whereas the Australian sample contained 31.96% missing values. Data were missing due to item nonresponse and Little (1988) Missing Completely at Random (MCAR) test was not significant, *x*^2^ (656) = 657.447, *p* = 0.477, indicating that the missing data did not show a systematic patten and are likely MCAR. Multiple imputation (Enders and Gottschall, 2011) was therefore used to handle missing data in the main analyses, as it allows for robust estimates by imputing plausible values for missing data under the assumption that the data were missing at random. First, the theoretically driven confirmatory factor analysis (CFA) was employed to test the fit of the factor structure of each scale separately for the two country groups. Once the factorial structure was validated using CFA, the pattern of relationships among the latent variables was tested via structural equation modeling (SEM) procedure in each country group.

Measurement invariance was then tested using multi-group confirmatory factor analysis (MGCFA) with progressively restrictive models. First, a configural model with no equality constraints was estimated. Next, metric invariance was assessed by constraining factor loadings to be equal across groups. Scalar invariance was then tested by additionally constraining item intercepts. Changes in RMSEA (ΔRMSEA) were used to evaluate model comparisons, with values ≥0.015 indicating meaningful deterioration in fit (Chen, 2007). Following the establishment of measurement invariance, structural invariance was assessed by imposing equality constraints on significant structural paths across groups. The Wald test of parameter constraints (Wang and Wang, 2020) was further conducted to evaluate cross-group differences in specific path coefficients.

The relatively small Australian sample (*N* = 97) may have implications for the robustness of the analyses. Specifically, when conducting CFA, the number of free parameters exceeded the sample size, as indicated by Mplus. To address this issue, the shortened 12-item version of the TSES was adopted. Another challenge was the unbalanced sample size between these two country groups. When conducing factorial invariance, the severely unbalanced sample sizes are more likely to reduce the power of detecting non-invariance as the large group has more weight in determining the final solution (Chen, 2007; Yoon and Lai, 2018). In the present study, the small Australian sample may therefore have limited the sensitivity of the analyses to detect potential non-invariance between the Chinese and Australian cohorts. In other words, the measurement invariance model is likely to be determined by the larger group. To address this potential issue, the data were reanalysed using a subsampling approach as recommended by Yoon and Lai (2018), with results indicating that it did not have a substantive impact on the findings (see details in appendix B).

#### 2.3.2 Qualitative analyses

Thematic analysis (Braun and Clarke, 2022) was used for qualitative data analysis in this research. The qualitative data were used specifically to contextualize and interpret the quantitative findings rather than to generate independent qualitative conclusions; accordingly, they are integrated within the discussion rather than presented as a separate results section (Plano Clark et al., 2010).

## 3 Results

### 3.1 Measurement invariance

Measurement invariance was tested in three stages: configural, metric, and scalar invariance. Table 2 presents the model fit indices for all the models tested (first-order model). Configural invariance (Model 1) tests whether the same factor structure holds across groups, meaning that the same pattern of fixed and free factor loadings is consistent. This step does not constrain any parameters to be equal across groups. Model 1 had good model-data fit (CFI = 0.962, TLI = 0.953, RMSEA = 0.049, and SRMR = 0.038) suggesting that the overall factor structure is consistent across the two groups. Metric invariance (Model 2) tests whether the factor loadings are equal across groups. This step ensures that the latent constructs are measured in the same way across the groups, allowing for comparisons of relationships between latent factors. Model 2 also had a good model fit (CFI = 0.961, TLI = 0.954, RMSEA = 0.048, and SRMR = 0.048) when tested for metric invariance. This additional constraint (set factor loadings equally) did not result in a meaningful difference between Model 1 and Model 2 (ΔRMSEA = 0.001), which provided support for the metric invariance between the two groups. Scalar invariance (Model 3) tests whether the intercepts of the indicators are equal across groups. This step is necessary to ensure that the latent variable means can be meaningfully compared between groups. Model 3 also provided sufficient fit for full scalar invariance (CFI = 0.950, TLI = 0.944, RMSEA = 0.053, and SRMR = 0.057). The difference in RMSEA was 0.005, suggesting that constraining the factor loadings and item intercepts equally across the Chinese and Australian sample did not significantly increased model misfit. These findings suggest that the underlying constructs are measured equivalently across the two groups, allowing for meaningful cross-cultural comparisons in subsequent analyses.

**Table 2:** Test of measurement invariance for the multi-group measurement model.

Model description		χ^2^	df	CFI	TLI	RMSEA	SRMR	Model	ΔCFI	ΔTLI	ΔRMSEA	ΔSRMR
CFA-CH (*N* = 527)		248.443 *p* < 0.001	124	0.966	0.958	0.044 0.036 0.051	0.033					
SEM-CH (*N* = 527)		378.353 *p* < 0.001	169	0.948	0.936	0.048 0.042 0.055	0.068					
CFA-AU (*N* = 97)		170.046 *SD* = 11.187	125	0.940 *SD* = 0.015	0.927 *SD* = 0.018	0.060 *SD* = 0.008	0.058 *SD* = 0.003					
SEM-AU (*N* = 97)		222.930 *SD* = 12.301	170	0.933 *SD* = 0.015	0.919 *SD* = 0.019	0.056 *SD* = 0.007	0.074 *SD* = 0.003					
M1	configural (*N* = 624)	432.581	249	0.962	0.953	0.049	0.038					
		*SD* = 9.363		*SD* = 0.002	*SD* = 0.002	*SD* = 0.001	*SD* = 0.001					
M2	Metric (*N* = 624)	451.369	262	0.961	0.954	0.048	0.048	2 vs 1	0.001	0.001	0.001	0.010
		*SD* = 9.38		*SD* = 0.002	*SD* = 0.002	*SD* = 0.001	*SD* = 0.001					
M3	Scalar (*N* = 624)	514.888	275	0.950	0.944	0.053	0.057	3 vs 2	0.011	0.010	0.005	0.009
		*SD* = 9.689		*SD* = 0.002	*SD* = 0.002	*SD* = 0.001	*SD* = 0.002					
M4	SEM with MI (*N* = 624)	708.707 *SD* = 11.047	365	0.934 *SD* = 0.002	0.925 *SD* = 0.002	0.055 *SD* = 0.001	0.078 *SD* = 0.001					
M5	SEM with MI and SI (*N* = 624)	715.179 *SD* = 11.085	370	0.933 *SD* = 0.002	0.925 *SD* = 0.002	0.055 *SD* = 0.001	0.079 *SD* = 0.001	5 vs 4	0.001	0.001	0.000	0.001

### 3.2 Structure invariance

Regarding the second question, the structural model was first tested separately in China and Australia. Table 3 presents mean, and standard deviations for the latent factors measured in both the Chinese and Australian samples. Specifically, Perceived university support, Perceived placement school support and three background variables including (a) specific classroom management coursework (0 = no or 1 = yes); (b) experience of interactions with school-aged children outside the classroom (0 = no or 1 = yes); (c) satisfaction about the CM content provided in the ITE program (ranging from 1 = very dissatisfied to 4 = very satisfied), were used as predictors of self-efficacy in student engagement, self-efficacy in instructional strategies, and self-efficacy in classroom management.

**Table 3:** Descriptive statistics of latent factors.

Latent Factors	Country	*N*	Mean	*SD*
Efficacy in student engagement	China	527	6.053	1.202
	Australia	88	6.411	1.053
Efficacy in instructional strategies	China	527	6.380	1.159
	Australia	88	6.703	1.028
Efficacy in classroom management	China	527	6.321	1.187
	Australia	88	6.435	1.188
Perceived university support	China	527	2.972	0.731
	Australia	77	2.957	0.661
Perceived placement school support	China	527	3.087	0.702
	Australia	81	3.374	0.544

**Figure 1:**
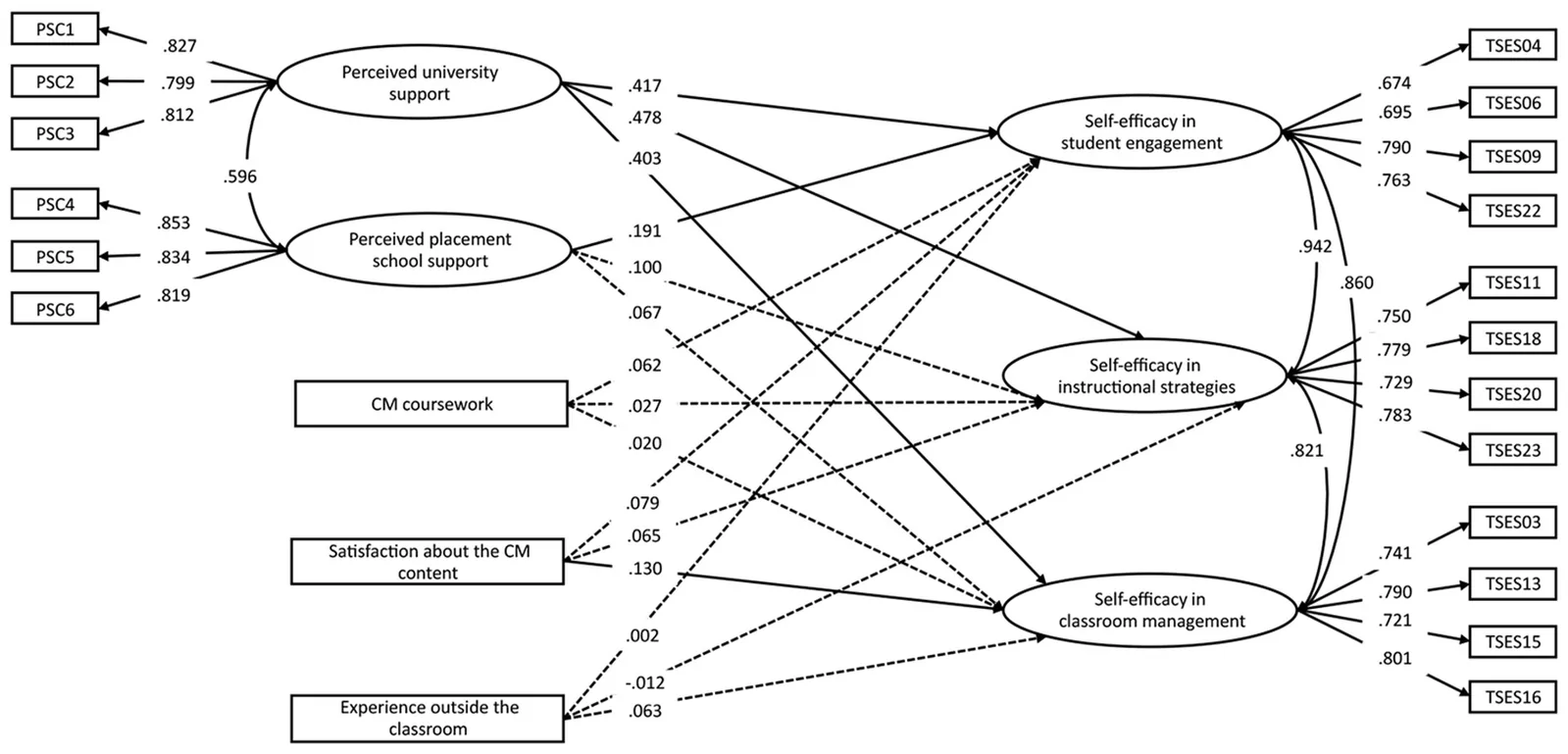
Structural model for the Chinese sample (*N* = 527). Standardized path estimates are reported, with non-significant paths shown as dashed lines and significant paths as solid lines.

**Figure 2:**
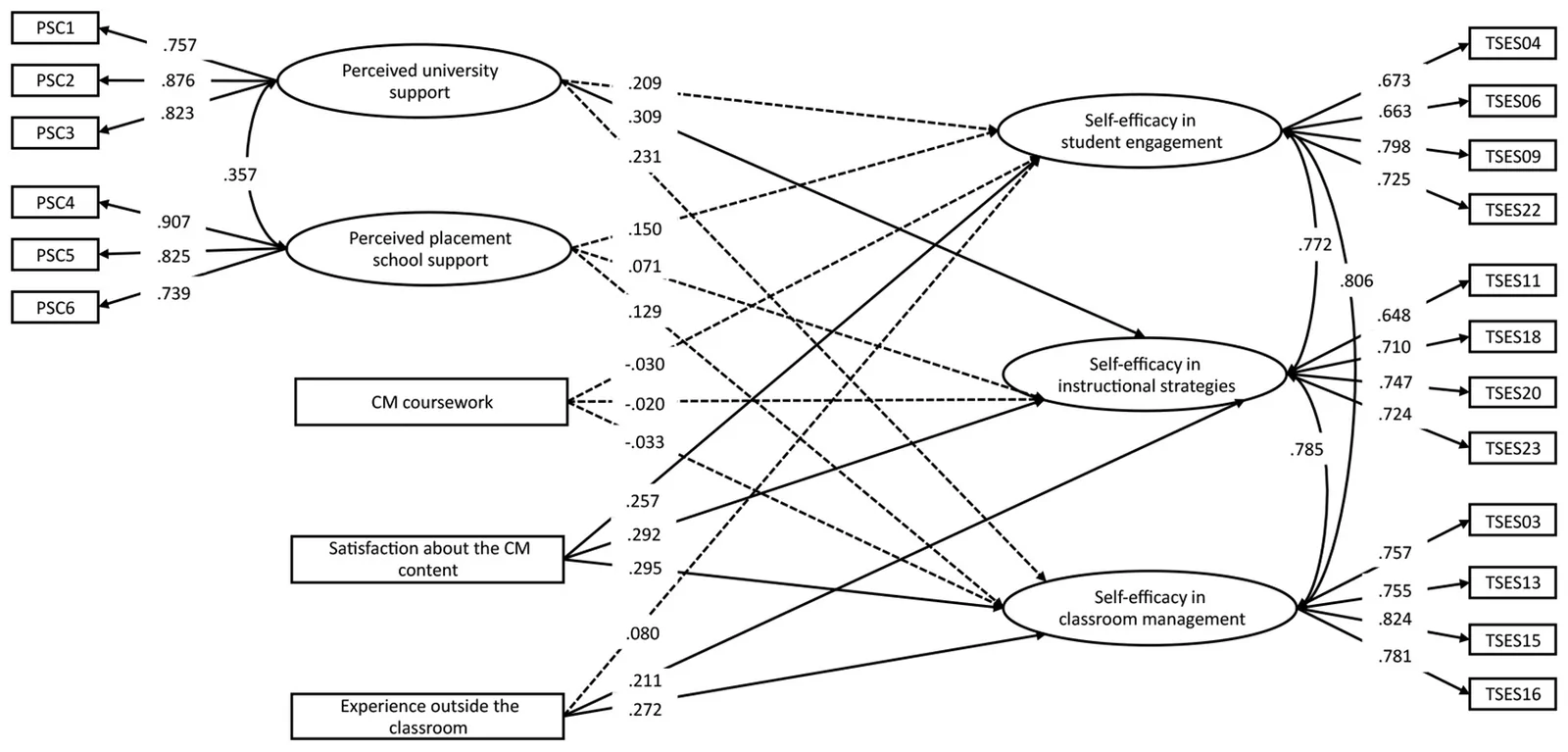
Structural model for the Australian sample (*N* = 97). Standardized path estimates are reported, with non-significant paths shown as dashed lines and significant paths as solid lines.

As shown in Table 2, both structural models for the Chinese and Australian samples demonstrated good overall model fit, indicating that the hypothesized relationships between the latent variables were well-supported in both groups. The results of the structural models for Chinese and Australian are presented in Figures 1 and 2. The estimated path coefficients differed to some extent between the two models, suggesting potential variations in how certain predictors influence the outcome variables across the two cultural contexts.

To formally test for structural invariance, constraints were imposed on the structural paths related to self-efficacy in classroom management. The results for the models were reported as follows (see Table 2): without constraints (Model 4), χ^2^ (365) = 708.707, CFI = 0.934, TLI = 0.925, RMSEA = 0.055, and SRMR = 0.078, and with constraints (Model 5), χ^2^ (370) = 715.179, CFI = 0.933, TLI = 0.925, RMSEA = 0.055, and SRMR = 0.079. Compared to Model 4, Model 5 did not result in a meaningful difference (ΔRMSEA <0.001). These results suggest that applying the invariance constraints across the groups did not significantly degrade the model fit.

In addition to the model comparison, the Wald tests (Wang and Wang, 2020) were also performed to identify whether specific path was invariant across the two groups. The Wald test allows for a more focused examination of individual paths, providing insight into whether specific relationships between variables differ significantly between the Chinese and Australian PSTs. The results of the Wald tests indicated that all five structural paths were invariant across the two groups (see Table 4). This means that the strength and direction of these key relationships did not differ significantly between the Chinese and Australian samples, supporting the conclusion that the structural model operates similarly in both groups. However, the path from experience outside the classroom to CMSE reached marginal significance (p=0.065), suggesting that some variations may exist in this relationship.

**Table 4:** Difference testing on path of regression between groups.

Structural path	Chinese	Australian	χ^2^	*P*-Value
Perceived university support → CMSM	0.400^***^	0.235	1.032	0.3096
Perceived placement school support → CMSE	0.068	0.131	0.230	0.6315
CM Course → CMSE	0.021	−0.047	0.307	0.5796
Satisfaction about the CM content → CMSE	0.130^**^	0.289^*^	1.231	0.2672
Experience outside the classroom → CMSE	0.063	0.278^**^	3.397	0.0653

^*^p < 0.05, ^**^p < 0.01, ^***^p < 0.001.

## 4 Discussion and implications

Regarding the first question, full metric invariance was achieved using MGCFA, and the subsampling approach provided additional support, indicating that the measured constructs have the same meaning across both groups. This allows for valid comparisons of the relationships between the latent construct and other variables between the two country groups. While the quantitative analysis did not reveal any significant differences between these two country groups, it provided valuable insights into the predictors of CMSE within each group. Understanding these predictors helps to shed light on how CMSE is shaped within specific educational and cultural environments, offering important implications for ITE programs in both China and Australia.

In the following sections, integration through narrative (Fetters et al., 2013) were used to merge the quantitative and qualitative results to draw integrated results. Specifically, we will present the quantitative results in response to the second research question, followed by the corresponding qualitative findings, and discuss how the qualitative results illustrate and expand on the quantitative data.

### 4.1 Personality

Due to poor reliability, the data of BFI-10 were excluded from the analysis, preventing the examination of the relationship between personality traits and CMSE. Other studies have also encountered similar issues when using the BFI-10. For example, Franz et al. (2022) investigated how PSTs' personality traits associated with their graduation and TSE, where personality traits were measured by the BFI-10. Although their study included a relatively large sample size (*n* = 5520), the short scale still yielded only moderate reliability, with one dimension excluded due to a low Cronbach's alpha. Future research could benefit from using longer scales for the Big Five to yield more robust results. Accordingly, any personality-related interpretations in the present study should be understood as exploratory and as primarily derived from the qualitative interview data, rather than from quantitative evidence based on the BFI-10.

Although quantitative data failed to examine the association between PSTs' personality traits and their CMSE, insights gathered from the interview data offered a deeper understanding of how personal factors might influence CMSE. For example, one participant (Australian-2) struggled with receiving positive feedback from mentors but still felt she could have achieved a more positive outcome for the student. She reflected, “*I do tend to over think things. I do set very high standards on myself”*, suggesting her personal characteristics influenced her perceptions of the events. This pattern of overthinking and self-criticism appeared to undermine PSTs' confidence and lead to burnout if left unchecked. This finding aligned with previous research (Morris and Usher, 2011; Morris et al., 2017; Shen, 2009). PSTs who employed positive coping strategies, such as reframing challenges or seeking social support, were able to mitigate the potentially adverse effects of stress and anxiety on their sense of self-efficacy. Conversely, those without established coping mechanisms may continuously feel that they fell short, even when performing well. Therefore, ITE programs may consider incorporating adaptive strategies to help PSTs build resilience.

No direct effects of PSTs' general behavioral tendencies on CMSE were found in this research although reference to personal traits were mentioned, e.g., “…*I may be a bit impatient…*” (Chinese-4), and “…*I'm not naturally an authoritative person…*” (Au- 9). While some personality traits such as high extraversion might aid in building rapport with students given its association with strong communication skills and effective interpersonal interactions (Barrick and Mount, 1991), PSTs in this sample did not perceive these personality characteristics as indicators of their CM capability. Instead, these behavioral tendencies are more likely to subtly shape PSTs‘ reactions to specific experiences, which may in turn impact their sense of self-efficacy.

### 4.2 Perceived support

The path *perceived university support*→*CMSE* is statistically significant for the Chinese group (β = 0.400^***^) but not for the Australian group (β = 0.235) in the MGCFA. Despite the difference in the strength and significance of these path coefficients, the Wald tests indicated that the underlying association between university support and CMSE operates similarly in both groups. The lack of statistical significance in the Australian group is likely due to its limited sample size (*n* = 97), which can reduce the statistical power of the analysis and make it more challenging to detect significant effects (Chen, 2022). The path coefficient for the Australian group (β = 0.235) still indicates a positive, moderate relationship between perceived university support and CMSE, suggesting that university support contributes positively to CMSE among Australian PSTs as well. This finding highlights the role of perceived university support as a factor positively associated with CMSE in both China and Australia.

The path *perceived placement school support*→*CMSE* is insignificant in both Chinese group (β = 0.068) and Australian group (β = 0.131), and the result of the Wald tests indicates that structurally, the relationship between perceived placement school support and CMSE is consistent across the Chinese and Australian groups. One possible interpretation of this non-significant relationship is that the effectiveness of placement school support may depend on the quality, consistency, and nature of the support provided by different placement schools and mentor teachers, rather than on perceived support more broadly. The interview data support this explanation. Some PSTs described their placement schools as highly supportive, with well-established systems that included regular feedback, mentorship from experienced teachers, and structured opportunities to develop CM skills. In contrast, others reported a lack of resources or inconsistent support, resulting in varying experiences across placement settings. These accounts were found in both country groups, which suggests that placement schools in both groups vary widely in their resources, mentorship structures, and commitment to supporting PSTs. To address this variability, ITE programs might consider building more consistent collaboration between universities and placement schools and implementing standardized guidelines for placement schools to ensure a minimum level of support across all placement schools.

Although perceived support is categorized as coming from the university or the placement school and quantitative data indicates these two sources play different roles in influencing CMSE, the interview data do not reveal a notable difference between university and placement school support. This lack of differentiation in the interview data may suggest that, from the perspective of PSTs, any form of support—whether from university or placement schools—contributes to their overall sense of self-efficacy in CM. Many PSTs described instances where support from mentors, university staff, or placement school colleagues provided reassurance and guidance during challenging situations, which helped them manage feelings of anxiety, self-doubt, and stress associated with CM tasks, e.g.,

“*The mentor's written feedback has some little emoji, which made me not feel so stressed out when I saw it*”

             (Australia-7).

Shen (2009) found perceived interpersonal support has effects on teachers' coping strategies. It seems that while personal factors may indirectly impact PSTs' CMSE, perceived support appears to interact with these factors, potentially mitigating negative personal influences on CMSE. For instance, strong interpersonal support can help buffer negative affective states and help build positive coping strategies. ITE programs, therefore, should prioritize robust support networks to enhance PSTs' resilience and adaptability, equipping them to handle classroom challenges confidently.

While no cross-cultural variations were found in terms of the effect of perceived support, Australian and Chinese PSTs appear to perceive varying levels of support during the placement. The descriptive statistics revealed that the Australian group (*M* = 3.087) perceived more support from their placement school than the Chinese group (*M* = 3.374). This finding was supported by the interview data. When asking participants how they felt about the support from their placement school, most Australian participants mentioned:

“*She's [mentor teacher] always welcomed [me] to approach [her]” (Australian-1); “I felt really supported”*

*             (Australian-8)*

“*He [mentor] gave me a lot of encouragements”*

*             (Australian-3)*.

. Whereas, most Chinese participants reported having minimal or even no interactions with their mentor teachers during their placements:

“*I don't feel very close to my mentor teachers”*

*             (Chinese-5)*

“*There weren't many interactions with him [mentor teacher] in general”*

*             (Chinese-9)*.

While this may be partly explained by cultural differences in the workplace—where Chinese PSTs may place greater value on authority figures and Australian PSTs tend to have more egalitarian relationships with their mentors—the mentorship experience reported by Chinese PSTs still appears to be lacking in quality interactions with their mentor teachers.

### 4.3 University-based coursework experience

The path *CM course*→*CMSE* is insignificant in both the Chinese (β = 0.021) and Australian (β = −0.047) groups in the MGCFA and the result of the Wald tests indicates that structurally, the relationship between taking a CM course and CMSE is consistent across the Chinese and Australian groups. Thus, taking a CM course was not a statistically significant predictor of PSTs' CMSE in the present quantitative model. This is of interest given that reviews of ITE courses around the world indicates a greater focus on CM is required (Davis, 2018; Montague and Kwok, 2022; Xu, 2015). One possible interpretation is that simply completing a CM course may not be sufficient to strengthen PSTs' CMSE, particularly if course content is not closely aligned with PSTs' practical CM needs. During the interviews, complaints about the CM content in ITE programs and suggestions for improvement were frequently discussed across both country groups, which may help explain why no significant relationship was found between taking specific CM courses and CMSE in either group. Given the findings in this research, it would indicate that any current courses are not meeting PSTs' expectations or requirements and therefore any future CM curricula should consider carefully both evidence-based research and PSTs' recommendations.

Specifically, PSTs' concerns included the limited CM content provided in their coursework, doubts about whether the CM knowledge gained at the university could be effectively applied in real classroom settings, and the qualifications and experience of their lecturers. According to Montague and Kwok (2022), CM content provided in ITE programs is inconsistent as teacher educators tend to teach CM related to their own comfort level (Stewart-Wells, 2000), which leads to gaps in PSTs' understanding of effective CM depending on the program they attend and who their educators are. O'Neill and Stephenson (2012a) conducted a survey among primary PST educators in Australia and found that CM courses coordinated by academics with CM research interests typically included more instructional hours and covered a greater range of research-based theories/approaches/models than courses led by those academics without such research backgrounds. Teacher educators' limited teaching experience has also been highlighted as an area of concern (Jones, 2006), especially for today's classrooms with increasing diversity of students and the growing use of electronic devices.

The path *Satisfaction about the CM content*→*CMSE* is statistically significant in both Chinese group (β = 0.130^**^) and Australian group (β = 0.289^*^) in the MGCFA and the result of the Wald tests indicates that despite the different effect sizes, there was no significant difference in the structure of the relationship between satisfaction with CM content and CMSE across the two groups. PSTs who are satisfied with CM content may find the course material useful, applicable, and aligned with real classroom challenges, leading to increased CMSE. As discussed above, CM courses vary widely in quality, depth, and practical application. The mere presence of a CM course in the curriculum may be insufficient for building confidence in classroom management. Satisfaction, however, could reflect how well PSTs perceive the quality and relevance of the CM content, which is associated with their confidence in CM. ITE program designers may consider using PSTs' satisfaction with CM content as an indicator of course effectiveness. Regular feedback loops with PSTs on CM courses could help ensure that content remains engaging, relevant, and aligned with classroom realities, and as recommended previously assist in developing CM courses that have a positive effect on CMSE.

Course/learning satisfaction has been viewed as a learning outcome (Lee et al., 2023); that is, if PSTs are satisfied with the CM content, they may acquire valuable CM knowledge. From this perspective, PSTs' knowledge or perceived knowledge in CM may act as a foundation for building their self-efficacy in CM. Although it seems reasonable that teachers with high levels of subject content/pedagogy knowledge tend to have stronger TSE beliefs (Hammack and Ivey, 2017; Hull et al., 2016; Newton et al., 2012), the interview findings revealed a more complex interaction between CM knowledge and CMSE. Some participants expressed that course experience/acquiring knowledge helped them manage their classrooms, while some demonstrated an advanced understanding of CM yet still struggled with their CMSE. As one participant (Australian-5) indicated:

“*It was great to know all the things that I could use or having all the equipment with me in my brain. But I didn't really know if they fully worked or not without having used them in person in the classroom*.”

Knowledge related to CM is undoubtedly valuable, however, it does not necessarily translate directly into higher CMSE. This finding aligns with Marschall (2023), who founded that “all pre-service secondary mathematics teachers have strong subject knowledge, yet all experience doubts in their TSE (p. 3)”. From this perspective, viewing mastery of subject/pedagogical knowledge as a direct source of TSE (Bautista and Boone, 2015; Palmer, 2011, 2006; Phan and Locke, 2015) may be inappropriate.

### 4.4 Informal experience

Prior experience interacting with school-aged children outside the classroom positively impacts Australian PSTs' CMSE (β = 0.278^**^) but not for Chinese PSTs (β = 0.063). Despite differences in path coefficients and significance levels, the results of MGCFA and the Wald tests demonstrated structural invariance, indicating that this path (informal experience → CMSE) function similarly across these two country groups. The interview data confirmed that there were no cross-cultural differences in terms of the influence of informal experience on CMSE. During the interview, participants mentioned several kinds of informal experience: tutoring, babysitting, out of school hours care (OSHC), sports umpiring and looking after close relatives (such as siblings, nieces and nephews). Mixed responses were found in both country groups regarding the question of whether additional experiences contributed to their CMSE, but PSTs' perceptions of whether informal experience were echoed across the two country groups.

The interview findings revealed that informal experience might contribute to CMSE under certain conditions, particularly when PSTs perceive similarities between these informal experiences and actual CM tasks. For example,

“*The only difference between umpiring and standing in the classroom is I don't have a whistle. I could compare the two very easily*”

             (Ausralian-4).

On the other hand, participants also explained why they perceived those informal experiences as less valuable, which may provide the explanations for the insignificant association found in the Chinese group. They posited that informal contexts were different from the classroom context, and they have not recognized any transferable skills across these different contexts, for example,

“*When I'm tutoring, I just help them with their schoolwork. With my own nieces and nephews, I don't have to deliver instruction in front of them, so, I don't put up a teacher's stance. It just very relaxing.”*

             (Chinese-9)

In other words, the effectiveness of informal experience in building CMSE depends on PSTs' ability to connect these experiences to CM demands. This finding suggests that ITE programs might encourage PSTs to reflect on and integrate their informal experiences with school-aged children. For example, programs might consider offering targeted workshops or sessions that help PSTs bridge the gap between informal and formal teaching experiences, increasing the perceived relevance of their informal experiences in the classroom. Additionally, the interview data did not reveal a clear pattern regarding specific type of informal experience which might be viewed as more effective. Future research could further explore the specific types of informal experiences that most effectively contribute to CMSE and under what conditions these experiences become relevant.

In addition to having other practice experience outside the classroom, PSTs' prior ideas and beliefs before entering an ITE program has also raised concerns (Chan and Elliott, 2004; Horgan and Gardiner-Hyland, 2019). Specifically, the beliefs PSTs hold prior to entering their ITE programs can influence their views of teaching and CM (Rebmann et al., 2015; Tang et al., 2014). These prior beliefs are likely to interact with PSTs' informal experiences, potentially reinforcing misconceptions or incorrect beliefs. Therefore, it is important for teacher educators to understand these beliefs and help PSTs to examine their beliefs about student-teacher relationships, issues of power, control, democratic classroom structures, and student misbehaviours. For example, ITE programs may consider providing workshops in small groups with adequate time to discuss the important concepts in CM and encourage PSTs to engage in reflective practice on what has contributed to their current beliefs.

## 5 Conclusion

This study extends research on the development of CMSE by moving beyond Bandura's four sources framework and instead identifying a set of predictors grounded in PSTs' actual teacher education program experiences. Across both Chinese and Australian contexts, CMSE was less shaped by formal indicators such as participation in CM courses and more by how pre-service teachers interpret and evaluate their experiences. Specifically, perceived support from universities and placement schools emerged as a key mechanism through which negative emotional states were mitigated, thereby facilitating more positive efficacy appraisals. The significantly lower levels of perceived support during placements reported by Chinese PSTs further highlights the importance of structured and consistent support systems within ITE. Formal CM coursework alone is insufficient for developing CMSE. PSTs' criticisms regarding course quality help explain the lack of association between course participation and CMSE, while satisfaction with CM content appears more influential. Similarly, prior experience with school-aged students contributes to CMSE only when PSTs perceive clear connections between these experiences and CM tasks, underscoring the importance of cognitive appraisal in transforming experience into self-efficacy. Finally, qualitative insights indicate that individual differences, particularly in adaptive coping strategies, may shape how PSTs interpret negative self-efficacy information. Taken together, these findings shift the focus from exposure to experiences to the processes through which such experiences are interpreted and supported. By foregrounding predictors that are closely aligned with PSTs lived experiences, this research offers actionable recommendations for supporting the development of CMSE in diverse educational contexts.

## 6 Limitation

While this study provides valuable insights into predictors of PSTs' CMSE, it is important to recognize its limitations, which may affect the generalizability, interpretation, and application of the findings. First, this study adopted convenience sampling. Research participants cannot be representative of the final year PSTs in China and Australian and the finding cannot be generalized to the whole population. Next, although specific strategies, such as simplifying the structural equation model and employing a subsampling approach, were implemented to address the limited sample size in the Australian group, the unbalanced sample sizes between the two groups might still constrain the ability to detect non-invariances effectively. Therefore, it is important to acknowledge that the cross-cultural comparison results should be interpreted with caution. Finally, although the SEM model allowed us to examine theoretically specified pathways among variables, the cross-sectional nature of the survey data precludes causal inference. The structural paths should still be interpreted as theoretically informed associations rather than causal effects.

## Data Availability

The datasets presented in this article are not readily available because Data are not publicly available due to ethical and confidentiality restrictions under the agreements with participating universities in China and Australia. Requests to access the datasets should be directed to; kerry.bissaker@flinders.edu.au.
