# Supplementary material for: What predicts preservice teachers' classroom management self-efficacy? A cross-cultural comparison between China and Australia
Source: Front Psychol. 2026 Aug 12;17:1892257. doi: 10.3389/fpsyg.2026.1892257 (PMC13509030; doi:10.3389/fpsyg.2026.1892257)
Supplement: Supplementary file 1 [file Supplementary_file_1.docx]

# Appendix A：

There are three scales in the questionnaire: the Teachers’ Sense of self-efficacy Scale (TSES) (Tschannen-Moran & Hoy, 2001), the 10-item Big Five Inventory (BFI-10) (Rammstedt & John, 2007), and the perceived support scale (Weber et al., 2019). TSES is a nine-point Likert-type scale including three dimensions of TSE—instructional strategies (eight items), classroom management (eight items) and student engagement (eight items). BFI-10 is a five-point Likert-type scale including five personality traits: extraversion (two items); agreeableness (two items); conscientiousness (two items); neuroticism (two items); openness (two items). The perceived support scale is a four-point scale including two dimensions: perceived support from university (three items) and perceived support from placement school (three items). The reliability of TESE and perceived support scales (with three or more items) were assessed using Cronbach's alpha and McDonald's omega with 95% confidence intervals. Whereas Cronbach's alpha and Spearman-Brown coefficient, instead of McDonald's omega, were used for BFI-10 as each dimension included only two items. The reliability coefficients of the scales across Chinese and Australian samples are reported in Table 1 & Table 2.

The TSES had high internal consistency reliability (α＞0.9) among both the Chinese sample and Australian sample. The reliability for the three sub-scales ranges from strong (α＞0.7) for student engagement and instructional strategies component in the Australian sample, to very strong (α＞0.9) for student engagement, instructional strategies components collected from the Chinese cohort, and classroom management component from both cohorts of participants. The McDonald’s Omega values for the TSES and the three sub-scales closely mirrored the Cronbach’s Alpha values. The 95% confidence intervals for Cronbach’s Alpha and McDonald’s Omega were narrow for the TSES and the three sub-scales, indicating the reliability estimates were precise.

The two sub-scales of perceived support scale had strong internal consistency for both the Chinese and Australian samples (α＞0.7), with nearly identical results for Cronbach’s Alpha and McDonald’s Omega. For the two sub-scales of perceived support scale, the confidence intervals were again narrow, indicating precision in the reliability estimates.

For the two-item sub-scales (extraversion, agreeableness, conscientiousness, neuroticism; and, and openness), all the Cronbach's alpha were below 0.7, which indicates poor reliability. While each personality dimension containing only two items is likely to have poor values of Cronbach's alpha (Carciofo et al., 2016), the alpha value of -0.107 for agreeableness in the Australian sample and the alpha value of -0.412 for openness in the Australian sample were unacceptable. Similarly, the Spearman-Brown coefficients ranged from low to moderate across Chinese and Australian samples. The Australian sample showed negative Spearman-Brown coefficients for agreeableness and openness, indicating a problem with the reliability of the scale. Therefore, the data of BFI-10 were excluded from the subsequent analysis.

| Table 1 Reliability coefficients for the scales/sub-scales-China | | | | | | | | | | | | | |
| --- | --- | --- | --- | --- | --- | --- | --- | --- | --- | --- | --- | --- | --- |
| Scales/sub-scales | | Items | | Cronbach's  Alpha | | 95% CI | | McDonald's  Omega | | 95% CI | | Spearman-Brown  coefficients | |
| Student Engagement | | 8 | | 0.906 | | [0.886, 0.921] | | 0.907 | | [0.889, 0.922] | | N/A | |
| Instructional Strategies | | 8 | | 0.916 | | [0.899, 0.929] | | 0.916 | | [0.900, 0.929] | | N/A | |
| Classroom Management | | 8 | | 0.912 | | [0.896, 0.926] | | 0.913 | | [0.898, 0.927] | | N/A | |
| TSES | | 24 | | 0.964 | | [0.957, 0.970] | | 0.964 | | [0.957, 0.970] | | N/A | |
| University support | | 3 | | 0.853 | | [0.822, 0.877] | | 0.854 | | [0.824, 0.879] | | N/A | |
| School support | | 3 | | 0.873 | | [0.847, 0.896] | | 0.874 | | [0.844, 0.899] | | N/A | |
| Extraversion | | 2 | | 0.586 | | [0.499, 0.663] | | N/A | | N/A | | 0.590 | |
| Agreeableness | | 2 | | 0.174 | | [0.014, 0.314] | | N/A | | N/A | | 0.179 | |
| Conscientiousness | | 2 | | 0.415 | | [0.301, 0.512] | | N/A | | N/A | | 0.424 | |
| Neuroticism | | 2 | | 0.309 | | [0.142, 0.438] | | N/A | | N/A | | 0.309 | |
| Openness | | 2 | | 0.246 | | [0.098, 0.374] | | N/A | | N/A | | 0.258 | |
| Table 2 Reliability coefficients for the scales/sub-scales-Australia | | | | | | | | | | | | |  |
| Scales/sub-scales | Items | | Cronbach's  Alpha | | 95% CI | | McDonald's  Omega | | 95% CI | | Spearman-Brown  coefficients | |  |
| Student Engagement | 8 | | 0.848 | | [0.788, 0.888] | | 0.847 | | [0.779, 0.889] | | N/A | |  |
| Instructional Strategies | 8 | | 0.870 | | [0.817, 0.908] | | 0.868 | | [0.799, 0.908] | | N/A | |  |
| Classroom Management | 8 | | 0.905 | | [0.868, 0.932] | | 0.907 | | [0.870, 0.934] | | N/A | |  |
| TSES | 24 | | 0.951 | | [0.931, 0.964] | | 0.950 | | [0.927, 0.963] | | N/A | |  |
| University support | 3 | | 0.851 | | [0.778, 0.897] | | 0.851 | | [0.786, 0.901] | | N/A | |  |
| School support | 3 | | 0.835 | | [0.738, 0.906] | | 0.838 | | [0.755, 0.906] | | N/A | |  |
| Extraversion | 2 | | 0.680 | | [0.505, 0.797] | | N/A | | N/A | | 0.681 | |  |
| Agreeableness | 2 | | -0.107 | | [-0.700, 0.308] | | N/A | | N/A | | -0.110 | |  |
| Conscientiousness | 2 | | 0.441 | | [0.126, 0.649] | | N/A | | N/A | | 0.458 | |  |
| Neuroticism | 2 | | 0.379 | | [-0.019,0.630] | | N/A | | N/A | | 0.379 | |  |
| Openness | 2 | | -0.412 | | [-1.235, 0.073] | | N/A | | N/A | | -0.427 | |  |

# Appendix B:

Yoon and Lai (2018) suggested that the statistical power to detect violations of measurement invariance may decrease when there are substantial differences in the sample sizes between the groups being compared. Given the unbalanced sample sizes in this research, with 527 participants in the Australian group and 97 participants in the Chinese group, this potential issue was taken into consideration. To address this, the data were reanalysed using a subsampling approach as recommended by Yoon and Lai (2018).

Specifically, a subset of the sample (n=97) in the larger group (Chinese group) was randomly selected 100 times to match with the smaller group (Australian group). The 100 random datasets were generated with the aid of python. Then, the measurement invariance analysis was completed 100 times for these 100 datasets including both Chinese (n=97) and Australian sample (n=97). These datasets were analysed in Mplus with metric and scalar invariance models using Robust Maximum likelihood (MLR) estimation. 200 Mplus input files (100 metric invariance models and 100 scalar invariance models) were also generated with the aid of python. To process these 200 Mplus input files, windows batch commands were used. Finally, the mean of each model fit statistic across the 100 datasets was calculated. Model fit statistics (model Chi-square, CFI, TLI, RMSEA, and SRMR) were extracted from each Mplus output file with the aid of python.

For the metric invariance model, the mean values of the fit statistics were as follows: 𝜒^2^ (263) = 367.341, CFI = 0.934, TLI = 0.924, RMSEA = 0.063, and SRMR = 0.076. These values indicate an acceptable model fit, suggesting that the factor loadings were comparable across the Chinese and Australian samples. This supports the conclusion that metric invariance holds, allowing for valid comparisons of relationships between the latent variables across the two groups.

However, the results of scalar invariance model, 𝜒^2^ (276) = 412.977, CFI = 0.914, TLI = 0.905, RMSEA = 0.071, and SRMR = 0.085 indicated an unacceptable model fit. Particularly given the decrease in CFI and TLI and the increase in RMSEA and SRMR beyond acceptable thresholds. The poor fit of the scalar invariance model suggests that the intercepts of the observed variables may not be equivalent across the two groups, meaning that latent means cannot be reliably compared between the Chinese and Australian preservice teachers.

Taken together, the metric invariance, which is considered as a minimal condition for measurement invariance, has been supported. In this condition, the relationships between latent variables and external variables were comparable across groups. However, the scalar invariance was not supported, which implies that caution should be taken with the results of latent factors mean comparison.
